# Trueness and Internal Fit of 3D Printed Provisional Veneers: An In Vitro Comparative Study

**DOI:** 10.3390/bioengineering11121204

**Published:** 2024-11-28

**Authors:** Anca-Elena Anghel-Lorinți, Andrei-Bogdan Faur, Raul N. Rotar, Anca Jivănescu

**Affiliations:** 1 Department of Prosthodontics, University of Medicine and Pharmacy “Victor Babes”, B-dul Revolutiei 1989, No. 9, 300580 Timisoara, Romania; anca.anghel@umft.ro (A.-E.A.-L.); rotar.raul@umft.ro (R.N.R.); jivanescu.anca@umft.ro (A.J.); 2 TADERP Research Center, Department of Prosthodontics, University of Medicine and Pharmacy “Victor Babes”, B-dul Revolutiei 1989, No 9, 300580 Timisoara, Romania

**Keywords:** intraoral scanner, laboratory scanner, internal and marginal fit, printed veneers, 3D printing

## Abstract

Dentistry is steadily evolving along the digital pathway at a constant and sure pace. Intraoral scanners (IOSs) started to enhance the precision and trueness of the restorations, making prosthodontics treatment more predictable. The objective of this study was to compare the trueness and internal fit of the printed provisional veneers for 60 preparations with three different types of finish lines. The abutments were scanned with the same intraoral scanner, and the resulting meshes were overlapped and analyzed in metrology software. The comparison was executed using two methods: (1) CAD (computer-aided design) designs vs. scanned veneers and (2) inverted surfaces of the preparations vs. scanned veneers. The butt joint preparation had the best trueness out of the three groups of preparation during both comparison methods, although there was no statistically significant difference between the butt joint and feather edge groups in the CAD design vs. scanned veneers analysis. Also, there was no statistically significant difference between palatal chamfer and feather edge groups in the scanned veneers vs. inverted surfaces of the preparations analysis.

## 1. Introduction

During the last few decades, dentistry has witnessed a high demand for computer-aided design and manufacturing (CAM/CAM) systems, all based on the rapid advancements in computer technology. The CAD/CAM concept and technology were introduced to create highly accurate restorations with minimal errors, in contrast to more traditional methods such as handcrafting, which are prone to more errors [1,2]. These CAD/CAM systems are classified into chairside and laboratory types [3]. Recent studies have shown that CAD/CAM restorations can exhibit superior marginal fit compared to conventional manufactured indirect restorations [4,5,6]. Laminate veneers (LVs) can also be created using CAD/CAM technology, where an intraoral scanner (IOS) can capture the preparation, and the software will create the digital design, followed by either milling or 3D printing from a selected material [7].

When talking about tooth preparation for LVs, there are various incisal designs such as feather edge, butt joint, and palatal chamfer [8,9]. Feather edge preparation avoids overlapping the LVs on the incisal edge, while the butt joint and palatal chamfer extend over the incisal margin [10]. Although challenges are present when opting for 3D printed restorations such as extended printing time or erratic post-processing, there are some advantages such as enabling a more complex design with less used material [11,12,13,14].

The present in vitro study aimed to compare the trueness and internal fit in 3D printed provisional veneers manufactured from a ceramic-filled hybrid material on teeth preparations with feather edge, butt joint, and palatal chamfer designs.

## 2. Materials and Methods

**Figure 1:**
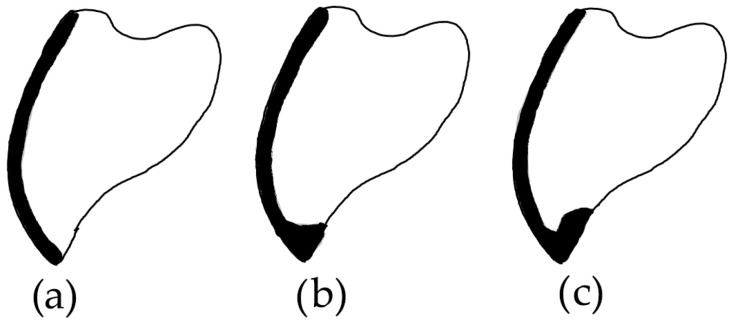
A schematic illustration of the three types of incisal margin design: (**a**) feather edge, (**b**) butt joint, and (**c**) palatal chamfer.

**Figure 2:**
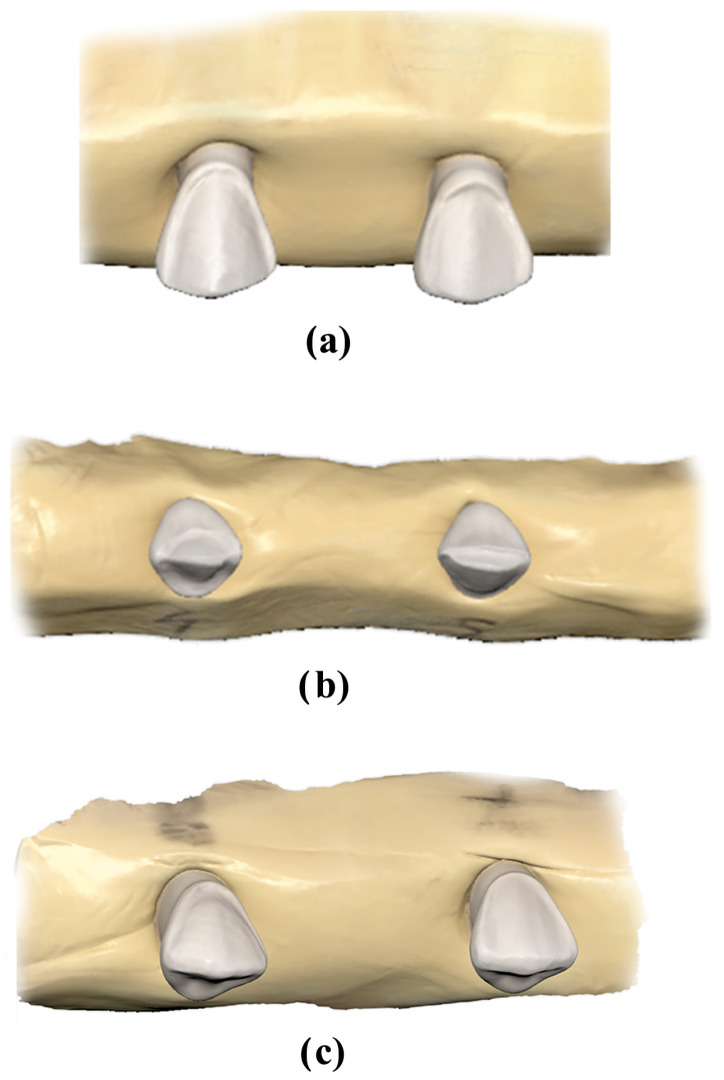
Digital models scanning of the upper right and left canines with different incisal preparation for veneers: (**a**) feather edge preparations, (**b**) palatal preparations, and (**c**) butt joint preparations.

Sixty LV preparations were performed on the upper right and left canine of a typodont (AG-3; Frasaco, Tettnang, Germany), consisting of 20 preparations for each of the three finish line types (feather edge, butt joint, palatal chamfer). The typodont was positioned in the mandibular joint of a dental mannequin (Phantom head PK-2 TSE; Frasaco, Tettnang, Germany). The preparations were then grouped based on the finish line type (Figure 1) and secured in putty polyvinylsiloxane (Elite HD, Zhermack, Italy) (Figure 2). An intraoral scanner (Medit i700, Medit Corp., Seoul, South Korea) was used for scanning the preparations, being previously calibrated according to the manufacturer’s indication. The Medit CAD app (Medit Corp., Seoul, South Korea) was used for designing the veneers, employing a 50 µm cement gap.

**Figure 3:**
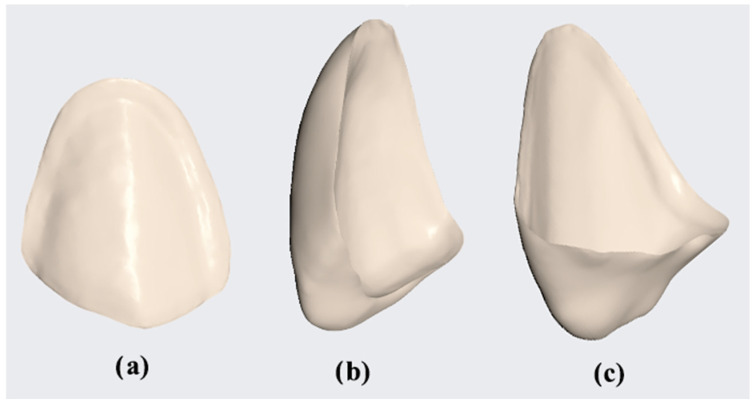
The design of the veneers: (**a**) feather edge, (**b**) butt joint, and (**c**) palatal chamfer.

Following the CAD, 60 provisional restorations were produced using a 3D printer (Prusa SL1, Prusa Research, Prague, Czech Republic) and SprintRay Crown resin (SprintRay, GmbH, Los Angeles, CA, USA) (Figure 3). The printer was calibrated according to the manufacturer’s instructions and resin’s instructions.

The same post-processing steps were used for all printed veneers (sandblasting with a glass bead blasting material 50 µm at a maximum blasting of 1.5 bar and curing for 6 min at 32.9 °C).

The next step was to scan the veneers with a laboratory scanner D700 3Shape (3Shape, Copenhagen, Denmark). The veneers were fixed in different points to simplify the scanning step of each inner face.

The CAD-designed meshes of the veneers were used as the reference models. Each scanned inner surface of the printed veneers was compared to the original CAD design file using metrology-grade quality control software (Geomagic Control X, Version: 16.0.2.16496, 3D Systems, Wilsonville, OR, USA). The area of interest was highlighted and marked as the reference data.

The inner surfaces of the printed restorations were imported into the Geomagic Control software and were submitted to the “Initial Alignment” step, continuing with the “Best Fit Alignment” to ensure an exact overlapping of the meshes.

**Figure 4:**
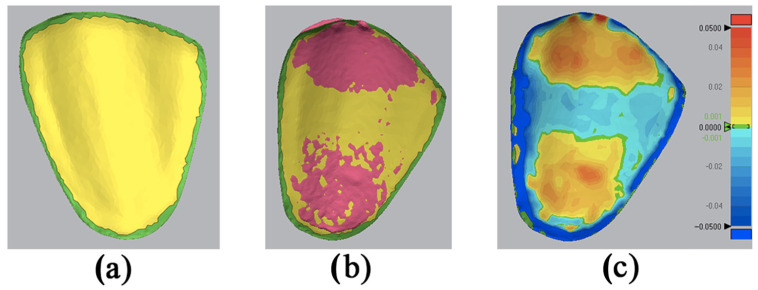
Different steps using Geomagic Control X metrology-grade quality control software: (**a**) reference data with the area of interest being distinguished, (**b**) layering of the IOS mesh over the reference mesh, and (**c**) a color-coded map showing the deviations of the intaglio surface.

Using the “3D Compare” function, all data points were paired onto the reference data, obtaining a standard deviation (SD) result between the measured and the reference. This step was a standard overlapping for all the samples to attain numerical figures of the SD. A color-coded map was generated in order to visualize the deviation patterns of the inner surfaces (±50 µm). The red spectrum showed an outward displacement, the blue spectrum demonstrated an inward displacement, and the green color indicated a no-deviation area (Figure 4).

A dedicated statistical program (MedCalc Software, West-Vlaanderen, Belgium) was used to sort and evaluate all the numerical data of the SDs. A parametric distribution for all the data was demonstrated by the Kolmogorov–Smirnov test for normality distribution. A one-way ANOVA test was implemented on all the data, carried out with a Newman–Keuls test. A relevance level of α = 0.05 was selected.

**Figure 5:**
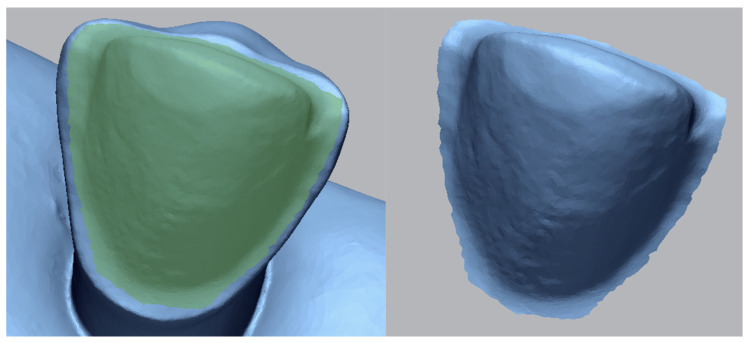
The digital model of the Frasaco tooth with the preparation. After using Geomagic software, every area of interest was saved as a STL file.

Next, the printed veneers were scanned with D700 3Shape. They were compared to the inverted scanned surfaces of the preparations. Using Geomagic, every area of interest was highlighted and saved as a .STL (standard tessellation language) file (Figure 5).

**Figure 6:**
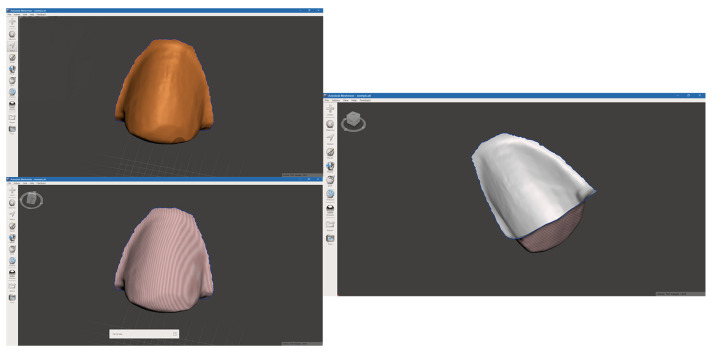
The STL file that resulted after inverting the mesh.

The STL file was imported in Autodesk Meshmixer Version 3.5 (Meshmixer, Autodesk, San Francisco, CA, USA), where the interested area was selected and inverted (Figure 6).

**Figure 7:**
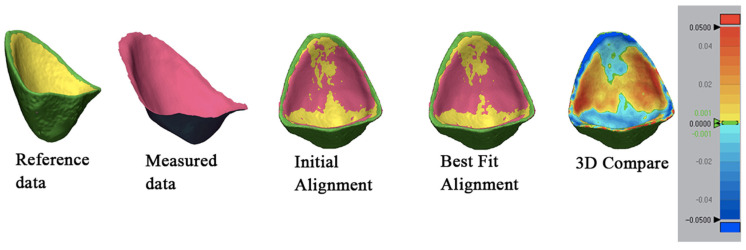
The Geomagic analysis of the inverted surfaces.

Each inverted surface was compared to the printed provisional veneer using the same metrology-grade quality control software and following the previously described steps (Figure 7).

Two groups were divided in order to determine the different statistical comparisons. In the first group, a comparison was made between the scanned veneers with the three types of preparation (feather edge, butt joint, and palatal chamfer preparations) and the CAD designs considered as the reference data. The second group compared the scanned veneers with the inverted surfaces of the preparations considered as the reference data. Both groups used the same statistical tests (Table 1).

A parametric distribution for all the data was demonstrated by the Kolmogorov–Smirnov test for normality. A one-way ANOVA test was implemented on all the data, carried out with a Newman–Keuls test. The relevance level of α = 0.05 was selected. The null hypothesis was that there would be no differences between the analyzed groups.

A post-hoc power analysis was performed to assess the adequacy of the study’s sample size in detecting statistically significant differences between groups. Given that a one-way ANOVA was employed to compare trueness across three finish line types, Cohen’s *f* was calculated as a measure of effect size. This was determined by calculating the between-group variance (*SSbetween*) and within-group variance (*SSwithin*) using group means, sample sizes, and standard deviations. Cohen’s f was computed as follows:

$$f=\sqrt{SSbetween/SSwithin}$$

With a significance level of 0.05, a sample size of 20 per group, and the calculated effect size, a post-hoc power analysis indicated that the achieved power was 1.0 for both comparisons. This result suggests that the study’s sample size was more than sufficient to detect the observed effects, supporting the reliability of the findings.

## 3. Results

In order to assess the statistical significance for the trueness and internal fit for every group, the root mean square (RMS) values were used in the statistical software MedCalc for the Kolmogorov–Smirnov test for normal distribution, and the one-way ANOVA test was carried out with the Student–Newman–Keuls test.

The first group (CAD designs and scanned veneers) rendered the following results (Table 2):

The analysis demonstrated a statistical difference between the groups, thus rejecting the null hypothesis (*p* < 0.001). The Newman–Keuls test presented differences (*p* < 0.05) between the CAD designs and scanned veneers. There was no significant statistical difference between the butt joint and feather edge groups. The palatal chamfer has shown an increased deviation with an average surface trueness of 86 μm. The butt joint and feather edge groups had similar deviations: 62.1 μm for the butt joint and 63 μm for the feather edge group.

The second group (scanned veneers-inverted surfaces of the preparations) had the following values (Table 3):

The analysis demonstrated that there is a statistical difference between the groups, thus rejecting the null hypothesis (*p* < 0.001). The Newman–Keuls test presented differences (*p* < 0.05) between the scanned veneers and the inverted surfaces of the preparations. There was no significant difference between the feather edge and palatal chamfer groups. The butt joint group has demonstrated a deviation with an average surface trueness of 37 μm. The feather edge and palatal chamfer groups had similar results: 52 μm for feather edge and 46 μm for palatal chamfer.

## 4. Discussion

The future of dentistry is starting to reshape in terms of restorative technologies, where all the laboratory work yielded by a dental technician is now elaborated upon using a digital protocol. This type of work is influencing the dentist and dental technician to get accustomed to operating larger volumes of digital data in a much cleaner environment, with finer details in a variety of different materials [15]. This alternative can contribute to creating trueness and accurate restorations for individual patients, with complex structures and quality standards similar to or better than the old-fashioned restorations [16,17,18,19,20]. Kessler et al. found that from a procedural and ecological point of view, subtractive production is limited by the smallest tool radius. Also, there is a limit to the number of objects that can be generated per machining operation [21].

At the same time, there are factors that can influence the quality of the intaglio surfaces of the veneers. The light curing can impact the polymerization shrinkage; during printing, a position pin can be too close to the margin of the veneer, resulting in a possible deformation during the detachment [22,23,24]. Alharbi et al. observed that “the build angle of 120 degrees combined with the thin support can offer the crown restoration the highest dimensional accuracy of the self-support surface area” [25].

Another limitation was the trueness and precision of the scanning machines, which were used according to the scanning distance [26]. Gavounelis et al. found an average trueness value below 50 μm and an average precision value below 5 μm for Medit i700, while Vafaee et al. reported a value of 47.27 μm and an average precision of 47.22 μm for the laboratory scanner D700, which confirmed the fact if the distance between the veneer and the scanner increased, the precision decreased [27,28]

Wadei et al. also reported “that the accuracy of 3D printed materials can be impacted by the type of printer, number of layers, layer thickness, printer wavelength, arrangement of crowns, UV intensity, total thickness, die-spacer thickness, post-processing method, and number or support structures” [29,30].

The right and left canines were selected for this study because they have high aesthetic and functional value and have one of the biggest probabilities of being treated with veneers [9,31]. When using the feather edge termination, only the unsupported enamel on the incisal margin can be removed (meaning the patient has a reliable occlusion) [32]. The butt joint has an overlap outline on the incisal edge [33]. Palatal chamfer incorporates the reduction of the incisal edge and a chamfer margin on the palatal surface [34].

Chaturvedi et al. asserted that if the restoration has a clear ending margin with an exact angle, that means the fit will be superior comparing the chamfer or feather edge margins, which is in line with the result of this study [35]. Wadei et al. confirmed that the increased marginal discrepancy is found in the chamfer finish line because it has a curved axio-gingival line angle, where errors can be found when 3D printing in layers [29].

When comparing the CAD design for the 60 preparations versus the scanned veneers, the butt joint finish line has the smallest standard deviation, meaning it has the best fit from all three groups. In terms of mean, the butt joint has the best result (62.1450 μm), second is the feather edge (63.0750 μm), and third is the palatal chamfer (86.4400 μm). But in terms of standard deviation, the butt joint comes first (±9.5492), the palatal chamfer second (±17.4220), and the feather edge third (±29.3514). From a statistical point of view, there is not a significant difference between the butt joint and feather edge groups, but both are significant to the palatal chamfer, which has the least trueness out of the three groups.

When analyzing the scanned veneers compared with the inverted surfaces of the preparations, in terms of mean, the butt joint has 37.7450 μm, followed by the palatal chamfer (46.0750 μm) and the feather edge (52.7300 μm). From the standard deviation standpoint, the butt joint group has ±8.1498, followed by the palatal chamfer group (±10.7433) and the feather edge group (±17.2830). From a statistical standpoint, there is not a significant difference between feather edge and palatal chamfer, but the butt joint group has the best trueness out of the three groups.

## 5. Conclusions

After analyzing and comparing 60 preparations, the three investigated preparation groups demonstrated distinctions regarding trueness and margin quality of the intaglio surfaces between CAD designs and the scanned veneers and between scanned veneers and the inverted surfaces of the preparations. This study makes the following conclusions:

(1) Butt joint preparation has the best trueness out of the three groups of preparation and the best trueness out of the two sets of analysis.
(2) There is no statistically significant difference between butt joint and feather edge groups in the CAD design-scanned veneers analysis.
(3) Regarding the scanned veneers-inverted surfaces of the typodont teeth preparations, there is no statistically significant difference between the palatal chamfer and feather edge groups.

## Figures and Tables

**Table 1:** Characteristics of the studied groups.

	Group 1	Group 2
Measured data	Scanned veneers (feather edge, butt joint, palatal chamfer) vs. CAD designs (feather edge, butt joint, palatal chamfer)	Scanned veneers (feather edge, butt joint, palatal chamfer) vs. Inverted surfaces of the preparations (feather edge, butt joint, palatal chamfer)
Reference data		

**Table 2:** Student–Newman–Keuls test for all pairwise comparisons. Mean and standard deviation of the inner surfaces (CAD designs-scanned veneers) trueness and margin values.

Group1 (Scanned Veneers-CAD Designs)	*n*	Mean	Standard Deviation	Different (*p* < 0.05) from Factor nr
(1) Butt joint	20	62.145 μm	9.549	(3)
(2) Feather edge	20	63.075 μm	29.351	(3)
(3) Palatal chamfer	20	86.440 μm	17.422	(1), (2)

**Table 3:** Student–Newman–Keuls test for all pairwise comparisons. Mean and Standard Deviation of the inner surfaces (scanned veneers-inverted surfaces of the preparations) trueness and margin values.

Group2 (Scanned Veneers-Inverted Surfaces of the Preparations)	*n*	Mean	Standard Deviation	Different (*p* < 0.05) from Factor nr
(1) Butt joint	20	37.745 μm	8.149	(2), (3)
(2) Feather edge	20	52.730 μm	17.283	(1)
(3) Palatal chamfer	20	46.075 μm	10.743	(1)

## Data Availability

All data are available upon request (Anca-Elena Anghel-Lorinți, A.E.A.L.; anca.anghel@umft.ro).
